# Cognability Across Adulthood: Neighborhoods and Cognitive Health Behaviors

**DOI:** 10.1093/geroni/igaf122.3283

**Published:** 2025-12-31

**Authors:** Grace Savard, Desiree Alvarez-McNelis, Mallory Sagehorn, Grace Bowman, Yue Sun, Michael Esposito, Jessica Finlay

**Affiliations:** University of Minnesota Twin Cities, Minneapolis, Minnesota, United States; University of Minnesota, Minneapolis, Minnesota, United States; University of Colorado Boulder, Boulder, Colorado, United States; University of Colorado Boulder, Boulder, Colorado, United States; University of Colorado Boulder, Boulder, Colorado, United States; University of Minnesota, Minneapolis, Minnesota, United States; University of Colorado Boulder, Boulder, Colorado, United States

## Abstract

While geographic variation in Alzheimer’s Disease rates suggests that environmental factors are important in the development of dementia, understanding of specific local contextual features that impact dementia risk across the life course is limited. This paper extends Cognability, a theoretical framework that conceptualizes how supportive a geographical area is for cognitive health through social and behavioral pathways, to a life course perspective. The Neighborhoods and Health at All Ages Study employed seated and mobile interviews (August 2023-March 2024) across the Minneapolis-St. Paul (MN) metropolitan area. Participants were on average 42 years old (range: 23-75). About half (53%) identified as female, 40% male, and 7% non-binary; 22% identified as Hispanic, 22% Asian, 18% non-Hispanic White, 17% Multiracial, 15% Black/African American, 3% American Indian/Alaska Native, and 3% Other. Reflexive thematic analysis identified ten key neighborhood sites that support cognitive health behaviors: parks and paths, recreation centers, eateries, grocery stores and markets, stores, civic/social organizations, religious organizations, arts and cultural amenities, libraries, and educational sites. More broadly, the study captured nuanced perspectives from socioeconomically, racially, ethnically, age, and gender diverse adults who uniquely experience and engage with combinations of amenities and hazards in their own neighborhoods. As the global dementia burden grows and disparities widen, our results can inform upstream community-level interventions to create more equitable neighborhoods that offer opportunities to support lifelong cognitive health and well-being.

